# Implementing pharmacogenetic testing in fluoropyrimidine-treated cancer patients: *DPYD* genotyping to guide chemotherapy dosing in Greece

**DOI:** 10.3389/fphar.2023.1248898

**Published:** 2023-09-14

**Authors:** Georgia Ragia, Anthi Maslarinou, Natalia Atzemian, Eirini Biziota, Triantafyllia Koukaki, Charalampia Ioannou, Ioanna Balgkouranidou, George Kolios, Stylianos Kakolyris, Nikolaos Xenidis, Kyriakos Amarantidis, Vangelis G. Manolopoulos

**Affiliations:** ^1^ Laboratory of Pharmacology, Medical School, Democritus University of Thrace, Alexandroupolis, Greece; ^2^ Individualised Medicine and Pharmacological Research Solutions Center (IMPReS), Alexandroupolis, Greece; ^3^ Department of Medical Oncology, University General Hospital of Alexandroupolis, Medical School, Democritus University of Thrace, Alexandroupolis, Greece; ^4^ Clinical Pharmacology Unit, Academic General Hospital of Alexandroupolis, Alexandroupolis, Greece

**Keywords:** *DPYD*, pharmacogenomics, fluoropyrimidines, 5-fluοrouracil, capecitabine, clinical implementation, polygenic algorithm, DPYD*TYMS*MTHFR interaction

## Abstract

**Introduction:** Dihydropyrimidine dehydrogenase (DPD), encoded by *DPYD* gene, is the rate-limiting enzyme responsible for fluoropyrimidine (FP) catabolism. *DPYD* gene variants seriously affect DPD activity and are well validated predictors of FP-associated toxicity. *DPYD* variants rs3918290, rs55886062, rs67376798, and rs75017182 are currently included in FP genetic-based dosing guidelines and are recommended for genotyping by the European Medicines Agency (EMA) before treatment initiation. In Greece, however, no data exist on *DPYD* genotyping. The aim of the present study was to analyze prevalence of *DPYD* rs3918290, rs55886062, rs67376798, rs75017182, and, additionally, rs1801160 variants, and assess their association with FP-induced toxicity in Greek cancer patients.

**Methods:** Study group consisted of 313 FP-treated cancer patients. *DPYD* genotyping was conducted on QuantStudio ™ 12K Flex Real-Time PCR System (ThermoFisher Scientific) using the TaqMan^®^ assays C__30633851_20 (rs3918290), C__11985548_10 (rs55886062), C__27530948_10 (rs67376798), C_104846637_10 (rs75017182) and C__11372171_10 (rs1801160).

**Results:** Any grade toxicity (1-4) was recorded in 208 patients (66.5%). Out of them, 25 patients (12%) experienced grade 3-4 toxicity. *DPYD* EMA recommended variants were detected in 9 patients (2.9%), all experiencing toxicity (*p* = 0.031, 100% specificity). This frequency was found increased in grade 3-4 toxicity cases (12%, *p* = 0.004, 97.9% specificity). *DPYD* deficiency increased the odds of grade 3-4 toxicity (OR: 6.493, *p* = 0.014) and of grade 1-4 gastrointestinal (OR: 13.990, *p* = 0.014), neurological (OR: 4.134, *p* = 0.040) and nutrition/metabolism (OR: 4.821, *p* = 0.035) toxicities. FP dose intensity was significantly reduced in *DPYD* deficient patients (*β* = −0.060, *p* <0.001). *DPYD* rs1801160 variant was not associated with FP-induced toxicity or dose intensity. Triple interaction of DPYD*TYMS*MTHFR was associated with grade 3-4 toxicity (OR: 3.725, *p* = 0.007).

**Conclusion:** Our findings confirm the clinical validity of *DPYD* reduced function alleles as risk factors for development of FP-associated toxicity in the Greek population. Pre-treatment *DPYD* genotyping should be implemented in clinical practice and guide FP dosing. DPYD*gene interactions merit further investigation as to their potential to increase the prognostic value of *DPYD* genotyping and improve safety of FP-based chemotherapy.

## 1 Introduction

The fluoropyrimidines (FPs) 5-fluorouracil (5-FU) and its oral prodrug capecitabine (CAP) constitute an efficient treatment of solid tumors. Despite their well-documented efficacy, toxicity is a serious drawback of FP therapy (Maslarinou et al., 2023). Toxicity rate in FP-treated patients has been reported to range between 10%–40%. Additionally, death rates due to FP-induced toxicity are relatively high. Per year, in France and the United States, respectively, approximately 150 and 1,300 FP-related deaths occur (Barin-Le Guellec et al., 2020; Deac et al., 2020).

Pharmacogenomics can be applied in FP therapy via the well-documented association of *DPYD* variations with FP-induced severe toxicity and dose requirements. *DPYD* encodes for dihydropyrimidine dehydrogenase (DPD), the rate limiting enzyme of 5-FU activation. To date, *DPYD* constitutes one of the most well characterized genetic loci. More than 80 *DPYD* variants have been identified and their functional effect on DPD activity has been characterized (Amstutz et al., 2018). Among them, DPYD*2A (rs3918290) and *13 (rs55886062) are the most common deleterious alleles leading to complete DPD loss of function (activity value 0), whereas c.2846T>A (rs67376798), and c.1129–5923C>G (rs75017182, HapB3) lead to decreased DPD activity (activity value 0.5). The released guidelines by Clinical Pharmacogenetics Implementation Consortium (CPIC) (Amstutz et al., 2018) and the Dutch Pharmacogenetics Working Group (DPWG) (Lunenburg et al., 2020) recommend FP dose reduction in *DPYD* defective patients ranging from 25% to 50% (activity score 0.5-1.5) or avoidance of FP use (activity score 0) (Amstutz et al., 2018; Lunenburg et al., 2020).

The benefits of *DPYD* genotyping towards reduction of FP-induced toxicity have been shown in a plethora of studies [reviewed in (Maslarinou et al., 2023)] with an established cost-effectiveness (Brooks et al., 2022; van der Wouden et al., 2022). Overall, results show that *DPYD* rs3918290, rs55886062, rs67376798, and rs75017182 variations are associated with increased toxicity risk and that this risk can be reduced when dose is genotype-adjusted without confronting therapeutic response (Maslarinou et al., 2023). Since April 2020, EMA recommends that “Patients should be tested for the lack of the enzyme DPD before starting cancer treatment with fluorouracil or with the related medicines, capecitabine and tegafur,” and that either phenotyping or genotyping can be used (EMA, 2020). Upfront *DPYD* genotyping of the four *DPYD* variants has been endorsed by several countries in Europe, including Germany, Austria, the Netherlands, Switzerland, the United Kingdom, Spain, and Italy (Martens et al., 2019; Wörmann et al., 2020; Begré et al., 2022; Etienne-Grimaldi et al., 2022; García-Alfonso et al., 2022; Wang et al., 2022; White et al., 2022; Bignucolo et al., 2023). In Italy, additionally to the four EMA recommended *DPYD* variants, rs1801160 (c.2194G>A, p.Val732Ile, DPYD*6) is also included in their ethnic dosing guidelines (Bignucolo et al., 2023). DPYD*6 is assigned by CPIC as a normal function allele, however, results of a recent meta-analysis show that DPYD*6 allele carriers have a 1.73-fold increased overall toxicity risk (Kim et al., 2022), therefore, in addition to the already known *DPYD* variants, *6 allele could be evaluated pre-emptively to reduce the risk of FP-induced toxicity (Del Re et al., 2019).

Despite the undisputable significance of *DPYD* variations on FP dosing decisions, for several countries, including Greece, data on the prevalence of *DPYD* variants and on their ethnicity-specific effect on FP-induced toxicity is still lacking. To fill this gap, the aim of the present study was to characterize the frequency of *DPYD* *2A, *13, c.2846T>A, HapB3, and *6 alleles in Greek cancer patients treated with the FPs 5-FU or CAP and to further analyze their association with FP-induced (severe) toxicity and dose intensity. Additionally, we have recently proposed the concept of a polygenic FP dosing algorithm (Maslarinou et al., 2023). Towards this direction, we have re-analyzed previous genetic associations found in the study population (Ioannou et al., 2021; Ioannou et al., 2022) in view of the presence of *DPYD* variations.

## 2 Patients and methods

### 2.1 Study population

Patient cohort has been described previously in detail (Ioannou et al., 2021; Ioannou et al., 2022). In brief, a total of 313 unrelated cancer patients (160 males and 153 females; mean age 64.2 years ± 10.6, range 34–88) treated with 5-FU or CAP in monotherapy or in combination with other antineoplastic drugs were included in the study. All patients were inpatients of the Department of Oncology of the Academic General Hospital of Alexandroupolis in Greece and were retrospectively enrolled from February 2018 to December 2019.

For each patient demographic and clinical data were recorded, including scheme dosage, duration of treatment and administration route, toxicity incidence and its effect on treatment. Toxicity was recorded by grade according to the common terminology criteria for adverse events v5.0 (National Cancer Institute (NCI), 2017). All patients were closely monitored by the same team of oncologists who were responsible for clinical decisions on chemotherapeutic regimen, dosages, timeline of drug administration and therapy discontinuation.

Informed written consent was obtained from each patient. The protocol of the study was approved by the Scientific Council and the Ethics Committee of the Academic General Hospital of Alexandroupolis (Greece) and was conducted according to the Declaration of Helsinki.

### 2.2 Genotyping

Approximately 3 mL blood was collected in EDTA tubes. Genomic DNA was extracted from peripheral whole blood using Gentra Puregene Blood Kit (QiagenR/Qiagen, MD, United States) or MagCore Automated Nucleic Acid Extractor (RBC Bioscience, New Taipei City, Taiwan), according to the instructions of the manufacturer, and stored at −20°C until use. DNA purity and quantity were assessed by UV-Vis Spectrophotometer Q5000 (Quawell) and Qubit 4 fluorometer (ThermoFisher Scientific), respectively.

*DPYD* genotyping was conducted in 96-well plates on QuantStudio™ 12K Flex Real-Time PCR System (ThermoFisher Scientific) using the pre-designed TaqMan^®^ allelic discrimination assays C__30633851_20 (rs3918290), C__11985548_10 (rs55886062), C__27530948_10 (rs67376798), C_104846637_10 (rs75017182) and C__11372171_10 (rs1801160) (ThermoFisher Scientific).

Each reaction was carried out in 10 μL of a total reaction volume containing 5 µL of TaqMan Genotyping Master Mix, 0.5 μL of 20x TaqMan assay and 4.5 μL (approximately 20 ng) of genomic DNA. PCR conditions for rs3918290, rs55886062, rs67376798, and rs1801160 were the following: 60 °C for 30 s (pre-read stage), 95 °C for 10 min, 50 cycles of 95 °C for 15 s and 60 °C for 1:30 min (PCR stage), and a final post-read stage at 60 °C for 30 s. For rs75017182, PCR conditions were identical except for 40 cycles of 95 °C for 15 s and 60 °C for 1 min in PCR stage. Non template controls were included in triplicates in each reaction. In 10% of randomly selected samples, genotyping was replicated by an independent researcher, with a 100% accordance in results.

For genotype calling, QuantStudio 12K Flex Software v1.5 was used. In the presence of at least one variant allele allelic discrimination plots were automatically generated, whereas in the absence of variations, genotype calling was based on multicomponent plots for each sample.

### 2.3 Statistical analysis

Shapiro–Wilk test was used to assess normality of continuous variables. Continuous variables are expressed as mean ± standard deviation (SD) in the case of normal distribution, otherwise they are expressed as median (25th, 75th percentiles). Based on normality, parametric (independent *t*-test or one-way ANOVA) and non-parametric (Mann–Whitney test or Kruskal–Wallis test) tests were used to compare continuous variables between two or more groups, as appropriate. Chi-square (χ^2^)-test or Fisher’s exact test was used for categorical data comparisons depending on number of observations. Allele frequencies were estimated by the gene counting method. Departure from Hardy–Weinberg equilibrium was estimated by an exact two-sided probability test using the formula provided by Weir (1996). True/False positive and negative cases were calculated to express sensitivity, specificity, positive (PPV) and negative (NPV) predictive values of *DPYD* genotyping in FP-induced any grade and grade 3-4 toxicity.

Logistic regression analysis with a stepwise forward selection procedure adjusted for sex, age, and weight was performed, with toxicity and severe toxicity incidence as the dependent variable and *DPYD* genotype or DPYD*other genes interaction as independent variable, to calculate odds ratio (OR). To estimate the likelihood of FP dose intensity associated with *DPYD* polymorphisms, beta coefficient (β) with 95% C.I. were calculated with a multivariable linear regression analysis with a stepwise forward selection procedure with FP dose intensity as a dependent variable and sex, age, weight, and *DPYD* deficiency as independent variables. A *p*-value less than 0.05 was considered statistically significant. The Statistical Package for Social Sciences for Windows, Version 27.0 (IBM Corp., NY, United States) was used for all analyses.

## 3 Results

### 3.1 Population characteristics

Patient demographic and clinical characteristics are presented in Table 1. The complete profile of patient clinicopathological data in pooled sample as well as stratified by sex has been previously described in detail (Ioannou et al., 2021; Ioannou et al., 2022). In terms of toxicity, FP-induced any grade (1-4) toxicity prevalence was 66.5% (208 patients) and the majority of adverse events (AEs) were gastrointestinal (37.1%). A total of 26 cases of grade 3-4 toxicity were recorded in 25 patients (12% prevalence within grade 1-4 toxicity). For a total of 79 patients, toxicity led to reduction of the administered dosage (*n* = 44), and/or delayed drug administration or therapy discontinuation (*n* = 59). Such adjustments were made in different chemotherapy cycles; *n* = 21 in cycle 1, *n* = 25 in cycle 2, *n* = 14 in cycle 3, *n* = 12 in cycle 4, *n* = 9 in cycle 5, *n* = 22 in cycle 6 and above.

**TABLE 1:** Main characteristics of patient cohort.

	FP-treated solid tumor patients (n = 313)
*Demographic characteristics*	
Sex (male, %)	160 (51.1)
Age, years (mean ± SD)	64.2 ± 10.6
Body Surface Area (median, 25, 75 percentiles)	1.79 (1.6, 1.9)
Smokers (n, %)	72 (23.0)
*Treatment characteristics*	
CAP-treated (n, %)	205 (65.5)
5-FU or CAP monotherapy (n, %)	10 (3.2)
5-FU and CAP (n, %)	55 (17.6)
XELOX (n, %)	144 (46)
CAP+oxaliplatin+MA (n, %)	41 (13.1)
CAP+oxaliplatin+taxanes (n, %)	34 (10.9)
CEF (n, %)	41 (13.1)
CMF (n, %)	17 (5.4)
FOLFOX or FOLFIRI (n, %)	16 (5.1)
5-FU+MA	3 (1.0)
5-FU+taxanes	4 (1.3)
*Response*	
Grade 1-4 toxicity (n, %)	208 (66.4)
Grade 3-4 toxicity (n, %)	26 (12.5)
Dose reduction (n, %)	44 (14)
Delayed drug administration or therapy discontinuation (n, %)	59 (18.8)
Relative dose intensity (median, 25, 75 percentiles)	100 (96, 100)

FP; fluoropyrimidine, SD; standard deviation, CAP; capecitabine, 5-FU; 5-fluorouracil, XELOX; capecitabine + oxaliplatin, MA; monoclonic antibody, CEF; 5-FU + epirubicin + cyclophosphamide, CMF; 5-FU + methotrexate + cyclophosphamide, FOLFOX; 5-FU + leucovorin + oxaliplatin, FOLFIRI; 5-FU + leucovorin + irinotecan + MA.

### 3.2 Frequency of *DPYD* polymorphisms

Among the four EMA-recommended *DPYD* variants, we have found 9 heterozygous carriers (2.9% prevalence); one patient carrying *2A deleterious allele (0.3%), and 8 patients carrying the reduced function rs75017182 allele (2.6%). Both *DPYD* *13 and rs67376798 alleles were absent from the studied population (95% C.I. 0.0001%–0.6%). *DPYD* rs1801160 polymorphism was more common in our population (42 heterozygous patients, 13.4%, and 1 homozygous patient, 0.3%) (Table 2).

**TABLE 2:** Prevalence of *DPYD* variants in total patient cohort and stratified per toxicity grade.

*DPYD* allele carriage	Total cohort (n = 313)	Grade 1-4 toxicity (n = 208)	*p*-value	Grade 3-4 (n = 25)	*p*-value
rs3918290 (*2A)	1 (0.3%)	1 (0.5%)	0.031	1 (4%)	0.004
rs75017182 (HapB3, c.1129-5923C>G)	8 (2.5%)	8 (3.8%)		2 (8%)	
rs55886062 (*13)	-	-		-	
rs67376798 (c.2846T>A)	-	-		-	
rs1801160 (*6)	43 (13.7%)	29 (13.9%)	0.775	2 (8%)	0.675
*2A-HapB3-*6 carriage	52 (16.6%)	38 (18.3%)	0.365	5 (20%)	0.567

### 3.3 Association of *DPYD* variants with FP-induced toxicity and dose intensity

All 9 patients carrying *DPYD* *2A or rs75017182 alleles experienced toxicity (*p* = 0.031). For grade 1-4 toxicity, PPV was 100%, NPV was 34.5%, with a 100% specificity and 4.3% sensitivity. *DPYD* deficiency increased odds of any grade gastrointestinal (OR: 13.99, 95% C.I. 1.71-114.22, *p* = 0.014), neurological (OR: 4.13, 95% C.I. 1.07-16.04, *p* = 0.040) and nutrition/metabolism (OR: 4.82, 95% C.I. 1.12-20.73, *p* = 0.035) toxicities.

In grade 3-4 toxicity cases prevalence of *DPYD* *2A or rs75017182 alleles was higher (3 out of 25 patients, 12.0%, *p* = 0.028) (PPV 33.3%, NPV 92.8%, specificity 97.9%, sensitivity 12%). *DPYD* deficiency increased odds of grade 3-4 toxicity in any system (OR: 6.49, 95% C.I. 1.45-29.06, *p* = 0.014). For gastrointestinal toxicity, *DPYD* defective variants significantly increased the odds of grade 3-4 toxicity (OR: 45.94, 95% C.I. 4.69-449.95, *p* = 0.001). Carriers of *DPYD* defective variants did not present with grade 3-4 toxicity in other systems.

In a model adjusted for sex, age, and weight, FP dose intensity was significantly reduced in *DPYD* deficient patients (*β* = −0.060, 95% C.I. −0.085, −0.035, *p* <0.001).

*DPYD* rs1801160 variant was not associated independently with FP-induced toxicity (*p* = 0.78 for any grade toxicity, *p* = 0.68 for grade 3-4 toxicity) or after adjusting for *DPYD* *2A and rs75017182 alleles (*p* = 0.77 for any grade toxicity, *p* = 0.51 for grade 3-4 toxicity). *DPYD* rs1801160 had no effect on dose intensity (*p* = 0.37).

### 3.4 Characteristics of patients carrying *DPYD* *2A and rs75017182 variants

Eight patients carrying *DPYD* *2A and rs75017182 variants, were treated with CAP-based combination chemotherapeutic scheme; 5 patients were on XELOX, and 3 patients on CAP+MA, whereas one patient was treated both with CAP or 5-FU based scheme (CAP or 5-FU +taxanes). The majority of patients (*n* = 7, 77.8%) had colorectal cancer. Eight out of nine *DPYD* variant carriers developed gastrointestinal toxicity. Oncologists proceeded to dose or therapy adjustment in 4 patients (5% of patients with dose or therapy adjustment). Dose was reduced only in the DPYD*2A carrier at the second cycle of chemotherapy due to grade 3-4 toxicity, as it is described in more detail in Section 3.5. For two patients who developed grade 1-2 toxicity, scheduled chemotherapy administration was delayed at cycle 4, whereas for the last carrier of *DPYD* variation, chemotherapeutic scheme was changed after first cycle.

### 3.5 Case report of the DPYD*2A carrier patient

In our study, one patient was carrier of the deleterious DPYD*2A allele having, thus, severely reduced DPD activity (activity score 1). This is the case of a 63 years old male colorectal cancer patient with pulmonary metastasis, treated with CAP at an initial daily dose of 3,500 mg. At the second cycle of chemotherapy, patient developed grade 1-2 diarrhea and CAP dose was reduced to 3,000 mg. At the third cycle, due to persisting diarrhea (grade 4) CAP dose was further reduced at 2,000 mg. At this dose, reduced by 43% of initially prescribed, patient did not experience any toxicity. According to CPIC dosing guidelines, the recommended starting dose for DPYD*2A heterozygous carriers is half of the standard dose. Prospective genotyping of this patient and subsequent *DPYD*-genotype CAP dose adjustment would have probably resulted in diarrhea avoidance and safe CAP treatment.

### 3.6 Interaction of *DPYD* *2A and rs75017182 variants with *TYMS* and *MTHFR* polymorphisms

In previous studies in the same patient cohort published by our team, we have found that *TYMS*-TSER 2R/2R genotype was associated with FP dose reduction due to toxicity in female patients (Ioannou et al., 2021) and that *MTHFR* 665C>T polymorphism increased both need for FP dose reduction (OR 5.05) and percentage of dose reduction (*β* = 3.318) again in female patients (Ioannou et al., 2022). We have herein further analyzed the interaction of DPYD*TYMS*MTHFR towards a polygenic prediction of FP-induced toxicity (Table 3). Neither *TYMS* nor *MTHFR* were independently associated in regression analysis with grade 3-4 toxicity in the pooled sample, however, DPYD*TYMS TSER 2R/2R and DPYD*MTHFR 665T+ interactions were associated with increased odds of severe toxicity (OR: 2.89, 95% C.I. 1.23-6.84, *p* = 0.015 and OR: 6.18, 95% C.I. 1.97-19.42, *p* = 0.002, respectively). Additionally, triple gene interaction DPYD*TYMS*MTHFR was associated with grade 3-4 toxicity (OR: 3.73, 95% C.I. 1.43-9.71, *p* = 0.007). No gene interactions were found for dose intensity (data not shown).

**TABLE 3:** Logistic regression analysis adjusted for sex, age and weight to estimate the odds of grade 3-4 toxicity in different genetic models.

Genetic model	OR	95% C.I.	*p*-value
***Predictive models***			
**Model A**			
DPYD*2A/rs75017182	6.49	1.45-29.06	0.014
Sex	1.42	0.60-3.38	0.43
Age	0.61	0.26-1.46	0.27
Weight	0.99	0.96-1.02	0.37
**Model B**			
DPYD*2A/rs75017182*TYMS TSER 2R/2R	2.89	1.23-6.84	0.015
Sex	1.49	0.63-3.56	0.37
Age	0.62	0.26-1.47	0.28
Weight	0.99	0.96-1.02	0.35
**Model C**			
DPYD*2A/rs75017182*MTHFR 665T+	6.18	1.97-19.42	0.002
Sex	1.56	0.64-3.76	0.33
Age	0.61	0.25-1.47	0.27
Weight	0.99	0.96-1.02	0.34
**Model D**			
DPYD*2A/rs75017182*TYMS TSER 2R/2R *MTHFR 665T+	3.73	1.43-9.71	0.007
Sex	1.52	0.63-3.65	0.35
Age	0.63	0.27-1.51	0.30
Weight	0.99	0.96-1.02	0.34
***Insignificant models***			
**Model A**			
DPYD*2A/rs75017182/rs1801160	1.43	0.50-4.04	0.51
Sex	1.48	0.63-3.47	0.37
Age	0.67	0.29-1.56	0.35
Weight	0.98	0.96-1.01	0.28
**Model B**			
*TYMS* TSER 2R/2R	1.15	0.48-2.78	0.76
Sex	1.47	0.63-3.44	0.38
Age	0.66	0.28-1.54	0.33
Weight	0.99	0.96-1.01	0.30
**Model C**			
*MTHFR* 665T+	1.12	0.49-2.56	0.80
Sex	1.47	0.62-3.44	0.38
Age	0.66	0.28-1.54	0.34
Weight	0.99	0.96-1.01	0.30

## 4 Discussion

In the present study we have genotyped a patient cohort consisting of 313 Greek cancer patients treated with the FPs 5-FU or CAP for five *DPYD* SNPs, namely, the EMA-recommended *2A, rs75017182, *13 and rs67376798, and, additionally, rs1801160. We have estimated the frequency of the four EMA-recommended polymorphism carriers at 2.9% and have found significant associations of *DPYD* variations with any grade FP-induced toxicity, severe toxicity and dose intensity. Analysis of *DPYD* rs1801160 SNP, did not improve the prediction models, albeit this SNP was higher in frequency (13.7% carriers). We have further applied a polygenic FP-induced toxicity prediction model in the study population and have found significant interactions among *DPYD*, *TYMS*, and *MTHFR* genes associated with grade 3-4 toxicity.

*DPYD* genotyping of *2A, rs75017182, *13, and rs67376798 constitutes the cornerstone of FP pharmacogenomics. Genotype-based FP dose reduction guided by these variations is currently implemented in several European countries prior to FP therapy initiation. However, to date, for Greece, data on *DPYD* variant frequency and on their effect on FP response in terms of toxicity is missing. In the studied population, we have detected heterozygosity of *2A and rs 75017182 variant alleles in nine patients. The *DPYD* deficiency frequency of 2.9% found in our study is consistent with the frequency of lower than 5% reported in European populations (Innocenti et al., 2020). For *13 and rs67376798 polymorphisms that were absent from the study population, the reported frequency in European populations ranges from 0% to 0.5%. *DPYD* rs1801160 polymorphism was more common in our population with an estimated allele frequency of 7% (13.7% carriers), in accordance with the frequency of approximately 5% reported in European populations (Kim et al., 2022).

DPYD*2A and rs75017182 polymorphisms were strongly associated with any grade toxicity; all patients carrying DPYD*2A and rs75017182 experienced FP-induced adverse events. In terms of severe (grade 3-4) toxicity, frequency of DPYD*2A and rs75017182 variants was increased (3 out of the 9 patients carrying these variants experienced severe toxicity). Both in any grade and in grade 3-4 toxicity, *DPYD* genotyping shows high specificity (100% and 97.9%, respectively). Sensitivity, however, is rather low ranging from 4.3% for any grade to 12% for grade 3-4 toxicity. Indeed, as described in other studies, the combined sensitivity of the major *DPYD* variants to predict 5-FU related toxicity is relatively low (5.3%), whereas specificity is above 99% (Lee et al., 2014). Therefore, in our study we confirm that in the Greek population *DPYD* genotyping of the EMA recommended polymorphisms has a prognostic value for FP-induced toxicity and can be used to select the optimal FP dose.

Additionally to the EMA-recommended *DPYD* polymorphisms, we have further analyzed *DPYD* rs1801160 variation. In our population, despite the rather high frequency of rs1801160 polymorphism, we found no association with FP-induced toxicity endpoints. *DPYD* rs1801160 variant is classified as a normal function allele, however, in different studies it was shown that after correcting for the four EMA-recommended alleles, rs1801160 increases risk of FP-induced toxicity (Del Re et al., 2019; Kim et al., 2022). The frequency of rs1801160 in Greek patient population is similar to the one reported in Europeans, however, adding rs1801160 in our prediction models decreased both PPV (from 100% to 72% for any grade toxicity) and specificity (from 100% to 86.7%). Our study could detect an effect of the magnitude reported in the meta-analysis by (Kim et al., 2022), if present in our population, with a power >75%. *DPYD* rs1801160 has not been associated straight forward with FP-induced toxicity in several studies (Toffoli et al., 2015; Varma et al., 2019), there are, however, reports showing that this variant allele may induce neutropenia risk (Ruzzo et al., 2017). In the recent meta-analysis by (Kim et al., 2022), rs1801160 shows an increased odds of neutropenia (OR 1.87) which is slightly increased to the odds of overall toxicity (OR 1.72). Therefore, in our study the lack of association could be hindered by the lack of records on levels of neutrophils. It should also be acknowledged that, in addition to *DPYD* genotype, other factors, notably microRNAs (Deac et al., 2021), have been shown to be predictive factors for FP toxicity; miR-27A is such a case. Interestingly, miR-27A rs895819 polymorphism was associated with FP-toxicity in *DPYD* variant allele carriers (Meulendijks et al., 2016). Thus, it cannot be excluded that *DPYD* rs1801160 effect is minor *per se*, but it can be masked by the presence of other variants. Such findings should be independently tested in different ethnic populations in order to establish the best fit genetic model to accurately predict patients who are at increased risk for FP-induced toxicity. Otherwise, implementing multiple variants without proven efficacy in a specific population endangers overestimating toxicity risk and underdosing of patients. It should be acknowledged that ethnicity is a significant contributor that should be taken into account as an adjusting factor for current genotype-based dosing algorithms. This has been shown for different pharmacogenomic based dosing algorithms, with *CYP2C9*/*VKORC1* pharmacogenomic dosing algorithm for vitamin K antagonists standing as a gold example (Ragia et al., 2017). Specifically for the FPs and *DPYD* variations, differences have been reported in Asian populations in whom *DPYD* variations have only a minor role in FP-related toxicity (Kanai et al., 2022).

*DPYD* undisputedly drives personalization of FP-dosing. However, a main limitation of *DPYD* polymorphisms is their rather low frequency resulting in low sensitivity. This fact highlights the need both to identify additional *DPYD* deleterious variants and to incorporate additional genes in dose prediction guidelines. *DPYD* is a well characterized genetic locus. It is known that additional, albeit rare variants, exist that may significantly increase FP-induced toxicity risk. Rare *DPYD* variants could contribute in predicting a larger fraction of DPD deficiencies and partially address the missing heritability and improve prediction of FP-induced toxicity (De Luca et al., 2022; De Mattia et al., 2022; Lešnjaković et al., 2023). It is expected, however, that even if additional rare variants are incorporated into *DPYD* dosing guidelines, sensitivity will remain low. We have recently discussed in detail that, beyond *DPYD*, several genes are involved FP-induced toxicity and that strong evidence exists that we are gradually moving from single *DPYD* based FP-dosing to a multigenic dosing approach (Maslarinou et al., 2023). Towards this direction, we have re-analyzed in our study population the association of *TYMS* and *MTHFR* gene polymorphisms in interaction with *DPYD* polymorphisms. We had previously shown that *TYMS* and *MTHFR* variations affect FP-response in a sex specific manner (Ioannou et al., 2021; Ioannou et al., 2022). In these studies, both *TYMS* and *MTHFR* were not independently associated with FP-induced toxicity. Interaction analyses show that, after correcting for *DPYD*, *TYMS* and *MTHFR* variations secondary increase FP-induced toxicity risk. Interestingly, the triple gene interaction DPYD*TYMS*MTHFR confers approximately a 4-fold increased odds of grade 3-4 toxicity, irrespectively of sex, while both 95% C.I. and *p*-value were improved compared to *DPYD* alone. *TYMS* and *MTHFR* encode for proteins that are not involved in 5-FU breakdown; these are thymidylate synthase, a target of FPs, and methylene tetrahydrofolate reductase that interferes with FP activity via thymidylate synthase inhibition (Maslarinou et al., 2023). The DPYD*TYMS*MTHFR interaction, thus, may be suggestive for additive effects of FP pharmacokinetic and pharmacodynamic variant factors and needs further investigation in larger patient cohorts. Ultimately, multi pharmaco-omics approach can and will be used in oncology (Ragia and Manolopoulos, 2022). It should be acknowledged that both machine learning and artificial intelligence models should be employed to classify the variants associated with FP-induced toxicity and construct tools for the clinical application of multigenic interactions (Shrestha et al., 2018; Maslarinou et al., 2023).

Our study has several strengths. Patient population is well characterized in terms of toxicity incidence. Additionally, all patients were closely monitored by the same small group of oncologists reducing thus variability in clinical decisions. All patients were previously genotyped for *TYMS* and *MTHFR* polymorphisms allowing therefore generation of gene interaction analyses. *DPYD* genotyping was performed by use of validated pre-designed TaqMan assays and 100% accordance in genotyping call was obtained by independent researchers. We should also acknowledge that unavoidable limitations exist in the study design. This is a retrospective study, therefore, the benefits of prospective *DPYD* genotyping in reducing FP-induced toxicity incidence cannot be assessed. Rare variants of *DPYD* that can shed additional light into toxicity mechanisms were not sequenced in our population. Additionally, in interaction analyses, other genes showing association with FP dosing requirements and toxicity incidence, such as *ENOSF1*, were not included. Patient chemotherapeutic schemes are FP-based, albeit it cannot be excluded that toxicity was induced by other agents. In our cohort, all *DPYD* variant carriers were treated with FP-based combination chemotherapeutic schemes; pharmacogenomic markers of other chemotherapeutic agents were not analyzed.

In conclusion, our study shows that approximately 3% of the Greek cancer patient population have reduced *DPYD* activity. We confirm the *DPYD* clinical implications in terms of FP-induced toxicity. *DPYD* *2A, rs75017182, *13 and rs67376798 based dosing can be applied in the Greek cancer population prior of FP therapy initiation. Towards a polygenic FP-dosing algorithm, interaction of DPYD*TYMS*MTHFR needs further validation in different populations.

## Data Availability

The original contributions presented in the study are included in the article, further inquiries can be directed to the corresponding authors.

## In memoriam

This paper is dedicated to the memory of Professor Nikolaos Xenidis (1966–2022).
